# Factors associated with non-invasive positive pressure ventilation failure in a COVID-19 intermediate care unit

**DOI:** 10.1186/s12245-023-00510-3

**Published:** 2023-05-12

**Authors:** Inês Farinha, Alexandra Tenda da Cunha, Ana Rita Nogueira, André Ribeiro, Carlos Silva, João Rua, João Trêpa, José Eduardo Mateus, Filipa Costa

**Affiliations:** 1 Pulmonology Department, Coimbra Hospital and University Centre, Praceta Prof. Mota Pinto, 3004-561 Coimbra, Portugal; 2 Intensive Care Medicine Department, Coimbra Hospital and University Centre, Praceta Prof. Mota Pinto, 3004-561 Coimbra, Portugal; 3 COVID-19 Intermediate Care Unit, Coimbra Hospital and University Centre, Praceta Prof. Mota Pinto, 3004-561 Coimbra, Portugal; 4 Haematology Department, Coimbra Hospital and University Centre, Praceta Prof. Mota Pinto, 3004-561 Coimbra, Portugal; 5 Internal Medicine Department, Coimbra Hospital and University Centre, Praceta Prof. Mota Pinto, 3004-561 Coimbra, Portugal; 6 Infectious Diseases Department, Coimbra Hospital and University Centre, Praceta Prof. Mota Pinto, 3004-561 Coimbra, Portugal

**Keywords:** Non-invasive ventilation, Respiratory insufficiency, COVID-19

## Abstract

**Background:**

The use of non-invasive positive pressure ventilation (NIPPV) in COVID-19 patients with hypoxaemia is still under debate. The aim was to evaluate the efficacy of NIPPV (CPAP, HELMET-CPAP or NIV) in COVID-19 patients treated in the dedicated COVID-19 Intermediate Care Unit of Coimbra Hospital and University Centre, Portugal, and to assess factors associated with NIPPV failure.

**Methods:**

Patients admitted from December 1st 2020 to February 28th 2021, treated with NIPPV due to COVID-19 were included. Failure was defined as orotracheal intubation (OTI) or death during hospital stay. Factors associated with NIPPV failure were included in a univariate binary logistic regression analysis; those with a significance level of *p* < 0.001 entered a multivariate logistic regression model.

**Results:**

A total of 163 patients were included, 64.4% were males (*n* = 105). The median age was 66 years (IQR 56–75). NIPPV failure was observed in 66 (40.5%) patients, 26 (39.4%) were intubated and 40 (60.6%) died during their hospital stay. The highest CRP (OR 1.164; 95%CI 1.036–1.308) and morphine use (OR 24.771; 95%CI 1.809–339.241) were identified as predictors of failure after applying multivariate logistic regression. Adherence to prone positioning (OR 0.109; 95%CI 0.017–0.700) and a higher value of the lowest platelet count during hospital stay (OR 0.977; 95%CI 0.960–0.994) were associated with a favorable outcome.

**Conclusions:**

NIPPV was successful in over half of patients. Highest CRP during hospital stay and morphine use were predictors of failure. Adherence to prone positioning and a higher value of the lowest platelet count during hospital stay were associated with a favourable outcome.

## Background

At the end of 2019, a novel coronavirus designated as severe acute respiratory syndrome coronavirus 2 (SARS-CoV-2) was identified in the city of Wuhan, China. SARS-CoV-2 disease (COVID-19) is a contagious disease that spreads mainly via droplet particles [1]. COVID-19 clinical manifestations may range from asymptomatic or mild disease, with flu-like symptoms, to critical disease, with severe pneumonia complicated by acute respiratory distress syndrome (ARDS) and acute respiratory failure [2–5].

Hypoxaemic COVID-19, present in 19–35% of cases, typically requires some form of respiratory support, the cornerstone being oxygen therapy [6, 7]. However, there is great debate on the optimal oxygenation and ventilation strategy in COVID-19 patients without indication for invasive mechanical ventilation (IMV) [2]. High-flow nasal oxygen (HFNO) was initially recommended as the first-line therapy for respiratory support in patients with hypoxaemia despite conventional oxygen, in detriment of non-invasive positive pressure ventilation (NIPPV), due to concerns about the possibility of particle aerosolization and contamination of the hospital staff [8, 9]. If HFNO is not available and there is no urgent indication for IMV, international guidelines suggest a NIPPV trial with close monitoring and short-interval assessment for worsening [10, 11]. The Portuguese Pulmonology Society issued a statement regarding respiratory non-invasive therapies in acute COVID-19 patients, suggesting that NIPPV might be used in negative pressure rooms, in patients with HFNO failure, or when HFNO is not available.

More recently, NIPPV has emerged as a valid therapeutic option to improve oxygenation and prevent orotracheal intubation (OTI). A European consensus document of management of acute respiratory failure associated with SARS-CoV-2 infection suggests continuous positive airway pressure (CPAP) use with helmet and without humidification (first choice), CPAP use with a oronasal mask (second choice) and non-invasive ventilation (NIV) use with an oronasal face mask (third choice) [12]. As a response to the large number of hospital admissions due to COVID-19 in Italy, the Italian Thoracic Society (ITS-AIPO) and the Italian Respiratory Society (IRS-SIP) proposed a protocol, which suggested the use of ventilatory support in dedicated COVID Units with close monitoring [13]. A study performed in Italy found the application of NIPPV (defined as HFNO, CPAP, and NIV) to be feasible and associated with improved outcomes, even though it was associated with a risk of staff infection [11].

The aim of this study is to evaluate the efficacy of NIPPV (CPAP, HELMET-CPAP, or NIV) in COVID-19 patients treated in a dedicated COVID-19 intermediate care unit and assess the factors associated with failure of NIPPV, defined as death or need for OTI.

## Methods

This is a retrospective observational study, performed in a dedicated COVID-19 Intermediate Care unit in Coimbra Hospital and University Centre, from December 1st 2020 to February 28th 2021. This 16-bed unit was meant for the treatment of more severe cases of COVID-19 infection and consisted of a fixed team of seven physicians working on rotating shifts. There was also a group of dedicated nurses with a nurse-patient ratio of 1:2 to 1:6. All patients were closely monitored 24 h a day via telemetry.

Adult patients with tachypnea (respiratory rate > 30 breaths per minute), respiratory effort, oxygen saturation below 90% on room air and haemodynamic instability due to SARS-CoV-2 infection (defined as a positive result on real-time reverse transcriptase–polymerase chain reaction assay of nasal and pharyngeal swab specimens) who required NIPPV and were admitted to a dedicated COVID-19 intermediate care unit were included in this study. Patients who were intubated less than 24 h after NIPPV initiation and who were still hospitalized at the time of data analysis were excluded.

Patients were treated with three different types of NIPPV: CPAP, NIV, and HELMET-CPAP. For the first 48 h, NIPPV support was practically permanent. Small breaks of 15 min were only allowed for meals and patients only switched to HFNO or conventional oxygen during those periods. Patients were usually placed in the prone position for as many hours a day as they could tolerate. After the first 48 h of NIPPV, if a clinical improvement was observed, the breaks from ventilation using HFNO or conventional oxygen were progressively increased. NIPPV withdrawal was determined when clinical stability criteria were met: respiratory rate below 20 cpm, with no respiratory effort, peripheral oxygen saturation (SpO2) above 96% and tidal volume below 7 mL/kg of ideal weight.

CPAP and NIV (V60 Plus®, Philips) were delivered by single circuit and an oronasal non-vented mask, with an anti-bacterial/viral filter between the interface and the exhalation port and another anti-bacterial/viral filter between the ventilator and the circuit. CPAP was started at 8 cmH2O and the continuous positive pressure was progressively increased 1 cmH2O to a maximal level of 15 cmH2O, to decrease patient respiratory rate, to reach the target tidal volume and to decrease ventilatory effort. Fraction of inspired oxygen (FiO2) was regulated for a SpO2 above 94%. NIV was used for patients who needed CPAP above 15 cmH2O, who experienced discomfort with CPAP or with hypercapnic respiratory failure or according to physician experience. Pressure support (PS) was regulated to reach a tidal volume of 6–7 mL/kg of ideal weight and to correct the hypercapnia. FiO2 was regulated for a peripheral oxygen saturation (SpO2) above 94%. HELMET-CPAP (StarMed Ventukit, Intersurgical) was delivered through a Venturi flow driver. FiO2 was set by regulating oxygen and airflow after connecting the oxygen source with the Venturi flow driver. Anti-bacterial/viral filters were applied to the expiratory port. PEEP and FiO2 were regulated to obtain a SpO2 above 94%.

Several data were collected from medical records: demographic information (age, sex); comorbidities; frailty; respiratory condition at admission (respiratory rate); blood sample exams during hospital stay; blood gas tests at admission, before and after 2–24 h of NIPPV initiation; time (days) from the beginning of symptoms to the beginning of NIPPV; ventilatory settings of the NIPPV; drugs administered during hospital stay. The PaO2/FiO2 ratio percentage change was calculated as: ((PaO2/FiO2 ratio during NIPPV-PaO2/FiO2 ratio during Venturi mask or reservoir mask)/PaO2/FiO2 ratio during Venturi mask or reservoir mask) × 100. Patients who died or underwent OTI were recorded. Informed consent was waived due to the retrospective nature of this study.

The primary outcome was NIPPV failure, defined as the occurrence of either OTI or death. Indication for OTI included the presence of the following criteria: inability to protect the airway; coma; life-threatening arrhythmias; severe hemodynamic instability (systolic blood pressure < 90 mmHg despite adequate fluid therapy or use of vasoactive agents); intolerance to NIPPV and progressive respiratory distress despite NIPPV optimization (respiratory rate above 30 breaths/min, tidal volume above 8 mL/kg of ideal weight, SpO2 below 94% and important respiratory effort). The presence of these criteria did not automatically imply OTI, since this decision was based on a multidisciplinary discussion. A Do-Not-Intubate (DNI) order was determined by the medical team in a case-by-case manner, based on concomitant comorbidities, functional status prior to SARS-CoV-2 infection, poor likelihood of survival and Frailty score. The assessment of factors associated with NIPPV failure was the secondary outcome.

Statistical analyses were performed using SPSS software, version 26.0 (IBM SPSS, Armonk, New York). Baseline characteristics of patients treated with different forms of NIPPV were compared. Descriptive statistics were described using absolute and relative frequencies for qualitative variables and mean, median and standard deviation (SD) for continuous variables. Categorical variables were compared using the Chi-squared test or Fisher’s exact test, as appropriate. Continuous variables were compared with the Mann–Whitney *U* and Kruskal–Wallis tests.

Factors associated with NIPPV failure were included in an univariate binary logistic regression analysis. Factors with a univariate significance level of *P* < 0.001 were selected to enter a multivariate binary logistic regression model and odds ratios (OR) with 95% confidence intervals (CI) were calculated for each factor. A value of *P* < 0.05 was considered statistically significant.

## Results

A total of 163 patients were included in the study, 64.4% were males (*n* = 105) and the median age was 66 years (IQR 56–75). Baseline characteristics of the study population are presented in Table 1. The most represented comorbidities were arterial hypertension (56.4%), obesity (33.7%), and type 2 diabetes (25.2%). The initial type of NIPPV was the following: 60.1% NIV (*n* = 98), 35.6% CPAP (*n* = 58), and 4.3% HELMET-CPAP (*n* = 7), with a mean (SD) initial FiO2 of 0.66 (0.20).

**Table 1:** Baseline demographic and clinical characteristics of the study population stratified by the type of initial NIPPV

	All		CPAP		NIV		HELMET-CPAP		*p* value
	*n* = 163 (100%)		*n* = 58 (35.6%)		*n* = 98 (60.1%)		*n* = 7 (4.3%)		
Age (years)–median (IQR)	66.0	(56–75)	63.5	(55–74)	67.5	(57–75)	48.0	(42–62)	0.013
Male sex–*n* (%)	105	(64.4)	38	(65.5)	64	(65.3)	3	(42.9)	0.505
Comorbidities–*n* (%)									
COPD	9	(5.5)	2	(3.4)	7	(7.1)	0	(0)	0.657
Asthma	12	(7.4)	4	(6.9)	8	(8.2)	0	(0)	1.000
Obesity	55	(33.7)	22	(37.9)	31	(31.6)	2	(28.6)	0.734
Diabetes	41	(25.2)	12	(20.7)	28	(28.6)	1	(14.3)	0.436
Coronary artery disease	14	(8.6)	5	(8.6)	9	(9.2)	0	(0)	1.000
Chronic heart failure	16	(9.8)	4	(6.9)	12	(12.2)	0	(0)	0.373
Arterial hypertension	92	(56.4)	29	(50.0)	61	(62.2)	2	(28.6)	0.114
Peripheral vascular disease	10	(6.1)	3	(5.2)	7	(7.1)	0	(0)	0.838
Stroke	5	(3.1)	3	(5.2)	2	(2.0)	0	(0)	0.489
Dementia	1	(0.6)	0	(0)	1	(1.0)	0	(0)	1.000
Solid cancer	2	(1.2)	1	(1.7)	1	(1.0)	0	(0)	1.000
Leukemia	2	(1.2)	0	(0)	2	(2.0)	0	(0)	0.569
Lymphoma	3	(1.8)	0	(0)	3	(3.1)	0	(0)	0.382
Chronic kidney disease	12	(7.4)	3	(5.2)	9	(9.2)	0	(0)	0.732
Peptic ulcer disease	5	(3.1)	2	(3.4)	3	(3.1)	0	(0)	1.000
Chronic hepatic disease	7	(4.3)	1	(1.7)	6	(6.1)	0	(0)	0.459
HIV	1	(0.6)	0	(0)	1	(1.0)	0	(0)	1.000
Connective tissue disease	3	(1.8)	2	(3.4)	1	(1.0)	0	(0)	0.611
Immunosuppressive therapy	5	(3.1)	2	(3.4)	3	(3.1)	0	(0)	1.000

*COPD* chronic obstructive pulmonary disease, *HIV* human immunodeficiency virus

Ninety-seven patients (59.5%) were successfully treated with NIPPV, while failure was observed in 66 (40.5%) patients: 26 (39.4%) were intubated and 40 (60.6%) died during hospital stay. Table 2 presents the clinical characteristics, laboratory, radiology findings, and medical therapy used in “NIPPV success” and “NIPPV failure” groups.

**Table 2:** Clinical characteristics, laboratory, radiology, and therapeutic data stratified by NIPPV failure

	NIPPV success		NIPPV failure		*p* value
	*n* = 97 (59.5%)		*n* = 66 (40.5%)		
Age (years)–median (IQR)	61.6	(52–61)	69.4	(62–76)	< 0.001
Male sex–*n* (%)	57	(58.8)	48	(72.7)	0.068
Scores at admission–mean ± SD					
SOFA	2.53	± 0.89	2.94	± 1.07	0.037
SIC	2.69	± 1.10	3.80	± 1.72	< 0.001
Frailty	2.63	± 0.68	3.30	± 1.14	0.001
Pulmonary infiltrates > 50% parenchyma on chest radiograph–*n* (%)	37	(38.1)	25	(37.9)	0.973
Blood tests–mean ± SD					
Highest value					
Creatinine (mg/dL)	1.13	± 1.62	2.58	± 3.14	< 0.001
LDH (U/L)	554.64	± 183.68	801.55	± 356.68	< 0.001
ALT (U/L)	95.14	± 82.38	94.24	± 126.11	0.066
Total bilirubin (mg/dL)	0.89	± 0.41	1.09	± 0.60	0.028
CRP (mg/dL)	15.83	± 8.05	25.24	± 10.90	< 0.001
Procalcitonin (ng/mL)	0.59	± 2.32	7.11	± 35.87	< 0.001
Ferritin (ng/mL)	1977.03	± 2059.99	3706.82	± 6039.27	0.001
Leukocyte count (G/L)	13.44	± 4.43	16.99	± 7.36	0.001
ESR (mm/h)	52.17	± 19.23	61.73	± 28.59	0.010
D-Dimer (ng/mL)	2664.70	± 6590	5056.32	± 9574	0.001
Lowest value					
Lymphocyte count (G/L)	0.70	± 0.34	1.02	± 3.05	< 0.001
Platelet count (G/L)	172.13	± 59.95	136.50	± 53.15	< 0.001
Arterial blood gas tests–mean ± SD					
PaO_2_ before starting NIPPV (mmHg)	82.18	± 36.95	81.05	± 45.76	0.137
PaCO_2_ before starting NIPPV (mmHg)	34.64	± 6.33	35.54	± 9.35	0.696
FiO_2_ before starting NIPPV	0.64	± 0.18	0.69	± 0.21	0.056
SpO_2_ before starting NIPPV (%)	93.85	± 3.94	91.90	± 5.69	0.061
Lactate before starting NIPPV (mmol/L)	1.80	± 0.70	1.93	± 0.93	0.637
PaO_2_ after starting NIPPV (mmHg)	121.91	± 44.51	103.65	± 55.98	< 0.001
PaCO_2_ after starting NIPPV (mmHg)	35.87	± 5.22	36.09	± 8.10	0.913
SpO_2_ after starting NIPPV (%)	97.27	± 1.71	95.59	± 3.62	< 0.001
Lactate after starting NIPPV (%)	1.76	± 0.55	1.80	± 0.65	0.865
PaO_2_/FiO_2_ ratio before starting NIPPV	138.25	± 69.42	125.62	± 68.13	0.038
PaO_2_/FiO_2_ ratio after starting NIPPV	189.04	± 77.26	130.29	± 70.32	< 0.001
PaO_2_/FiO_2_ ratio variation	55.21	± 75.34	15.55	± 57.72	< 0.001
NIPPV–mean ± SD					
Duration of NIPPV (days)	5.32	± 2.90	7.05	± 6.22	0.737
Duration of symptoms until starting NIPPV (days)	8.11	± 3.65	7.64	± 4.66	0.552
Door-to-mask time (days)	2.53	± 1.70	3.73	± 3.69	0.221
Highest IPAP (mmH_2_O)	16.57	± 2.46	18.57	± 2.49	< 0.001
Highest EPAP/CPAP (mmH_2_O)	12.36	± 1.34	13.48	± 1.35	< 0.001
Highest tidal volume (mL)	697.77	± 164.25	809.79	± 293.84	0.028
Highest respiratory rate (cpm)	27.58	± 5.17	35.44	± 9.40	< 0.001
Treatment–*n* (%)					
Steroids	97	(100.0)	62	(93.9)	0.025
Remdesivir	70	(72.2)	25	(37.9)	< 0.001
Antibiotics	31	(32.0)	40	(60.6)	< 0.001
Morphine	5	(5.2)	32	(48.5)	< 0.001
Sedation^a^	13	(13.4)	31	(47.7)	< 0.001
Adherence to prone positioning	83	(92.2)	25	(50.0)	< 0.001

*ALT* alanine aminotransferase, *CPAP* continuous positive airway pressure, *CRP* C-reactive protein, *EPAP* expiratory positive airway pressure, *ESR* erythrocyte sedimentation rate, *FiO2* fraction of inspired oxygen, *IPAP* inspiratory positive airway pressure, *IQR* interquartile range, *LDH* lactate dehydrogenase, *NIPPV* non-invasive positive pressure ventilation, *PaCO2* partial pressure of carbon dioxide, *PaO2* partial pressure of oxygen, *SD* standard deviation, *SIC* sepsis-induced coagulopathy, *SOFA* sequential organ failure assessment, *SpO2* oxygen saturation

^a^Other than morphine (benzodiazepines. antipsychotics)

Overall, the mean (SD) duration of NIPPV treatment was 6.0 (4.6) days. Dexamethasone and Remdesivir were the drugs most used for treatment, in 97.5% and 58.3% of patients, respectively. The overall in-hospital mortality was 33% (*n* = 54). Aggravating respiratory failure was the cause of death in 48 patients (88.9%), while other causes were identified in 6 patients, including nosocomial infections (*n* = 5) and cerebrovascular accident (*n* = 1). A DNI order was determined in 45 patients (27.6% of the entire sample), 86.7% of whom died. Among the patients who were candidates for OTI (*n* = 118), NIPPV was successful in 91 patients (77.1%). Twenty-six patients (16% of the entire sample) were intubated and transferred to the intensive care unit (ICU), 14 of whom died, accounting for a ICU mortality rate of 53.8%.

Factors associated with NIPPV failure with a value of *p* < 0.05 were selected and an univariate logistic regression analysis was performed for each of them (Table 3). Factors associated with NIPPV failure in the univariate analysis with a value of *p* < 0.001 were selected for a posterior multivariate logistic regression model. These included age, highest LDH, CRP or leukocyte count, lowest platelet count, arterial blood oxygen saturation 2–24 h after the beginning of NIPPV, maximal values of IPAP and EPAP/PEEP used, maximal respiratory rate, remdesivir, antibiotics or morphine use, need for sedation and adherence to prone positioning. The results of the multivariate analysis and the corresponding OR and 95%CI are presented in Table 4. Values of PaO2/FiO2 before and 2–24 h after starting NIPPV were not included in the multivariate logistic analysis to prevent problems of collinearity in the interpretation of the results.

**Table 3:** Results of the univariate logistic regression analysis

	OR	(95% CI)	*p* value
Age	1.058	(1.028–1.089)	< 0.001
Frailty score	2.479	(1.460–4.211)	0.001
Highest creatinine	1.590	(1.162–2.177)	0.004
Highest LDH	1.004	(1.002–1.006)	< 0.001
Highest total bilirubin	2.330	(1.15–4.721)	0.019
Highest CRP	1.114	(1.068–1.161)	< 0.001
Highest procalcitonin	1.328	(1.073–1.643)	0.009
Highest ferritin	1.000	(1.000–1.000)	0.010
Highest leukocyte count	1.111	(1.047–1.178)	< 0.001
Highest ESR	1.018	(1.001–1.036)	0.041
Highest D-Dimer	1.000	(1.000–1.000)	0.112
Lowest lymphocyte count	1.089	(0.912–1.300)	0.347
Lowest platelet count	0.988	(0.982–0.995)	< 0.001
PaO_2_ after starting NIPPV	0.992	(0.985–0.999)	0.026
SpO_2_ after starting NIPPV	0.741	(0.626–0.876)	< 0.001
PaO_2_/FiO_2_ ratio before starting NIPPV	0.997	(0.992–1.002)	0.258
PaO_2_/FiO_2_ ratio after starting NIPPV	0.987	(0.982–0.993)	< 0.001
Highest IPAP	1.380	(1.182–1.612)	< 0.001
Highest EPAP/CPAP	1.966	(1.469–2.630)	< 0.001
Highest tidal volume	1.002	(1.001–1.004)	0.006
Highest respiratory rate	1.192	(1.115–1.274)	< 0.001
Steroids	0.000	(0.000–?)	0.999
Remdesivir	0.235	(0.121–0.458)	< 0.001
Antibiotics	3.275	(1.705–6.291)	< 0.001
Morphine	17.318	(6.236–48.088)	< 0.001
Sedation^a^	5.891	(2.754–12.602)	< 0.001
Adherence to prone positioning	0.084	(0.033–0.218)	< 0.001

*CPAP* continuous positive airway pressure, *CRP* C-reactive protein, *EPAP* expiratory positive airway pressure, *FiO2* fraction of inspired oxygen, *IPAP* inspiratory positive airway pressure, *LDH* lactate dehydrogenase, *NIPPV* non-invasive positive pressure ventilation, *PaO2* partial pressure of oxygen, *SpO2* oxygen saturation

^a^Other than morphine (benzodiazepines. antipsychotics)

**Table 4:** Results of the multivariate logistic regression analysis

	OR	(95% CI)	*p* value
Age	0.996	(0.931–1.065)	0.905
Frailty score	0.412	(0.141–1.010)	0.105
Highest LDH	1.005	(1.000–1.010)	0.045
Highest CRP	1.164	(1.036–1.308)	0.011
Highest leukocyte count	1.102	(0.925–1.312)	0.277
Lowest platelet count	0.977	(0.960–0.994)	0.010
SpO_2_ after starting NIPPV	0.839	(0.562–1.251)	0.388
Highest IPAP	0.711	(0.424–1.495)	0.198
Highest EPAP/CPAP	2.636	(0.962–7.220)	0.059
Highest respiratory rate	1.006	(0.863–1.172)	0.940
Remdesivir	0.801	(0.170–3.765)	0.778
Antibiotics	0.864	(0.168–4.434)	0.861
Morphine	24.771	(1.809–339.241)	0.016
Sedation^a^	3.974	(0.539–29.302)	0.176
Adherence to prone positioning	0.109	(0.017–0.700)	0.020

*CPAP* continuous positive airway pressure, *CRP* C-reactive protein, *EPAP* expiratory positive airway pressure, *IPAP* inspiratory positive airway pressure, *LDH* lactate dehydrogenase, *NIPPV* non-invasive positive pressure ventilation, *SpO2* oxygen saturation

^a^Other than morphine (benzodiazepines. antipsychotics)

Among the selected factors, highest CRP during hospital stay (OR 1.164; 95%CI 1.036–1.308) and morphine use for management of respiratory distress or sedation (OR 3.974; 95%CI 0.539–29.302) were identified as independent predictors of OTI or in-hospital death after applying the multivariate logistic regression model. Adherence to prone positioning (OR 1.109; 95%CI 0.017–0.700) and a higher value of the lowest platelet count during hospital stay (OR 0.977; 95%CI 0.960–0.994) were associated with a favourable outcome.

## Discussion

This study aimed to investigate factors associated with NIPPV failure, including CPAP, HELMET-CPAP, and NIV, in COVID-19 patients treated in a dedicated intermediate care unit. NIPPV success was observed in 59.5% of patients with severe acute respiratory failure, suggesting it may be a valid therapeutic option in patients with acute respiratory failure associated with SARS-CoV-2 infection, in this setting. Highest CRP during hospital stay and morphine use were considered independent factors associated with NIPPV failure. Adherence to prone positioning and a higher value of the lowest platelet count during hospital stay were associated with a favourable outcome.

Considering NIPPV failure, some factors were significantly more associated with this outcome. Older age and Frailty score accounted for a higher dependency status. SIC score and highest level of D-Dimer were associated with a higher incidence of microvascular coagulopathy, commonly related to SARS-CoV-2 infection. The highest serum level of CRP, ESR, ferritin and leucocyte count were associated with NIPPV failure since they are linked to a higher inflammation rate typical of COVID-19 related pneumonia, showing there may be a connection between high inflammatory parameters and disease progression with worse NIPPV outcomes. Highest serum level of procalcitonin and lymphocyte count was probably associated with a higher incidence of bacterial superinfection. Highest serum level of creatinine accounted for a poorer kidney function. On the other hand, the association between the highest level of the lowest platelet count and NIPPV success may be related to the fact that thrombocytopenia is related to severe infection and worse outcomes.

Before the onset of NIPPV, our data suggests that respiratory failure was overall equal between the two groups. However, the response to the NIPPV (represented by PaO2, SpO2, and PaO2/FiO2 in the first arterial blood gas test after the start of this therapy) was significantly lower in the group who failed NIPPV. Regarding the NIPPV parameters and monitoring data, in this sample of patients, the highest value of IPAP, EPAP/CPAP, tidal volume, and respiratory rate was significantly greater in the NIPPV failure group. The increase in respiratory effort induced by higher positive airway pressures, tidal volumes and respiratory rates may account for the development of patient self-inflicted lung injury (P-SILI), a mechanism known to contribute for lung damage and a poor response to NIPPV.

As far as treatment is concerned, this is the first study indicating the use of morphine and non-adherence to prone positioning as having a significant and independent role in NIPPV failure. Morphine is an opiate which has been shown to reduce dyspnea without significant ventilatory depression in patients in respiratory distress. Here we hypothesize a potential downside of morphine use in this specific population: the possibility of the reduction in respiratory rate, which may aggravate hypoxaemic respiratory failure. Morphine-induced sedation and the use of other sedatives (benzodiazepines and antipsychotics) in agitated patients who are poorly adapted to NIPPV may also account for these results. Prone positioning is aimed to reduce mismatch in ventilation/perfusion and therefore it is considered particularly important in the management of patients in ARDS. In COVID-19 patients, it has been shown to be a safe and effective means of improving oxygenation. In this study, non-adherence to prone positioning, mainly attributed to difficulties in maintaining the position due to neck and thoracic pain or agitation, was associated with NIPPV failure. The fact that Remdesivir was significantly less administered in the NIPPV failure group may be due to a more prolonged disease before hospital admission, making this group of patients non-eligible for this treatment. Antibiotics were significantly more used in the NIPPV failure group, accounting for the presence of bacterial superinfection in these patients, a factor predicting a worse clinical evolution.

However, this study presents some limitations. One of them is its retrospective design. The information was entirely collected from medical records, therefore making the study more susceptible to relevant missing data. As an example, the authors were unable to evaluate the effect of respiratory rate in NIPPV failure. Additionally, there was no control group for comparison purposes. As far as laboratory parameters are concerned, the first arterial blood gas test after the initiation of NIPPV was performed within 2 to 24 h of ventilation, which may be considered a relatively long time period.

## Conclusions

This study reveals that NIPPV is a valid option to treat COVID-19 patients with acute respiratory failure in high dependency units. Highest CRP during hospital stay and morphine use are independent predictors of NIPPV failure. Adherence to prone positioning and a higher value of the lowest platelet count during hospital stay were considered protective. Further prospective studies are needed to validate these findings.

## Data Availability

The datasets used and/or analyzed during the current study are available from the corresponding author on reasonable request.
